# Energy Sovereignty: A Values-Based Conceptual Analysis

**DOI:** 10.1007/s11948-022-00409-x

**Published:** 2022-11-03

**Authors:** Cristian Timmermann, Eduardo Noboa

**Affiliations:** 1 Ethics of Medicine, Medical Faculty, University of Augsburg, Augsburg, Germany; 2 Sustainability and Climate Change, Universidad Andina Simón Bolivar, Quito, Ecuador

**Keywords:** Empowerment, Self-determination, Energy policy, Self-subsistence, Sustainability

## Abstract

Achieving energy sovereignty is increasingly gaining prominence as a goal in energy politics. The aim of this paper is to provide a conceptual analysis of this principle from an ethics and social justice perspective. We rely on the literature on food sovereignty to identify through a comparative analysis the elements energy sovereignty will most likely demand and thereafter distinguish the unique constituencies of the energy sector. The idea of energy sovereignty embraces a series of values, among which we identified: (i) accessibility, to allow access to everyone, (ii) empowerment and recognition, to develop and sustain capabilities to collaboratively produce solution-oriented energy system knowledge and effectively participate in governance, (iii) stewardship and sustainability, to be able to design and manage decentralised renewable systems in view of protecting the environment, (iv) self-sufficiency, to reduce the negative shocks of exploitative business practises, (v) resilience, to maintain production capacities while withstanding socioeconomic, political, environmental and climatic shocks, (vi) peace, to establish production systems that do not involve hostile relations, (vii) transparency and self-determination, to establish democratic decision-making mechanisms that give a voice to previously underrepresented groups and limit corporate takeover (viii) gender-justice, by acknowledging the contributions of women and eliminate barriers to their empowerment. With a conceptual framework of energy sovereignty, we present a rationale that draws on the key values to be considered when formulating policy solutions for the energy sector.

## Introduction

As an element of energy politics, the idea of achieving energy self-sufficiency and independence has already some tradition. These goals have been defended in terms of energy security to reduce dependency on foreign energy suppliers – many of which pursue aggressive political and military agendas – and as part of a commitment to mitigate climate change and the resulting political instability (Giddens, 2009). Increasingly we can observe the desire to take independent action, either individually or as a community, to define energy policies and produce energy locally under the concept of energy justice (Jenkins, 2019). This form of justice demands, among others, being able to decide as a community, a fair distribution of benefits and burdens, and recognition. To back these demands this discourse bases its demands on ideas of procedural justice, distributive justice and recognition justice (Jenkins et al., 2016; Pesch et al., 2017).

A similar discontent about production systems can be found in the agricultural sector. Farmers organisations around the world have voiced their demands under the concept of food sovereignty. As a matter of sovereignty, they seek to counter the dominance of seed giants and food retailers, to produce food in harmony with nature, and to maintain heritage varieties, among other demands (Via Campesina, 1996). These demands resemble those within the energy sector, craving for independence from fossil fuel giants, compliance with emission reduction goals, and more opportunities to develop autonomous systems. What can we learn about the food sovereignty movement for the energy sector? What are the benefits of framing demands for change as a matter of energy *sovereignty*? To gain a better understanding of the idea of energy sovereignty, we explore how the main findings and demands of food sovereignty advocacy can help us to sketch a preliminary conceptual framework on energy sovereignty, while keeping in mind both the fundamental differences and commonalities. As a first step we need to gain clarity on what the two underlying terms in “energy sovereignty” mean by themselves.

What is energy? Energy needs and wants vary with industrial development stage, proximity to human agglomerations, climatic, geographic and geological conditions, and food availability. The most widespread energy use is heating, principally for food preparation and maintaining bodily temperature. As industrial development increases, the energy demands grow exponentially, starting with the mechanisation of food production and conservation, to the transportation and the manufacture of complex materials. For the purposes of this analysis, we can talk about energy as the common denominator satisfying the most widespread needs.

What about sovereignty? To achieve sovereignty over something is to possess a sufficient degree of self-determination and non-domination. In its most basic form, sovereignty is the ability to exercise self-determination without others exerting undue interference (Gould, 2006). Sovereign nations may relinquish certain powers to guarantee freedom and limit matters of public concern (Rabotnikof, 2005). People who seek sovereignty must commit to certain positive duties, such as participating in decision-making and defending democratic institutions. For sovereignty to be universally achievable, people also have negative duties, such as not interfering in other governments (Kaul, 2010).

As a second step, we should ask ourselves: why do we need to discuss energy sovereignty? Increasing social complexity has made us dependent on energy. In today’s world, human rights, such as the right to food and the right to health, can only be widely secured with a well-functioning energy grid. Many food products and medicines spoil without refrigeration and some medical treatments need machines powered by electricity. At the same time, surveillance systems for natural disasters, disease outbreaks and crime prevention count on energy supply for their functioning and communication. This dependency on the energy system makes us vulnerable and demands that we as democratic societies retain control over production to avoid outside domination. Furthermore, as we cannot avoid consuming energy while functioning as a large-scale society, the manner in which energy is being produced and used is becoming increasingly important. To allow morally responsible consumption we need to drastically reduce the environmental footprint and the contribution to climate change (Heffron et al., 2015). We have a moral obligation to drastically reduce our environmental footprint not only for the sake of future generations, but to address the fundamental interests of younger generations (Zakaras, 2016). Here we find another similarity between the food and energy sector. In today’s world we are dependent on both and increasingly we cannot avoid being complicit as consumers for the harms our production systems cause.

We already possess the technology for a radical change. The technological feasibility of supplying energy cost-effectively from renewable resources for one's household is vastly different today than even a decade ago. New technological developments allow people to become energy producers, to monitor energy use and to organise themselves using information technologies. Yet to realise energy sovereignty people need to be empowered, by having access to the means of production and information, developing capabilities, and acquiring organisational skills. A democratisation of energy systems can be achieved through the diversification of energy production, where people, assisted by a process of local capacity building and accompanying regulatory frameworks, are able to make their energy decisions based on the resources available in their geographical area, their needs and the socio-environmental challenges they face (Thombs, 2019). Since people are already demanding self-determination in energy policies and the freedom to produce energy (Espe et al., 2018), it is urgent to examine the normative contents of energy sovereignty.

## Change in the Energy Sector

Energy sovereignty embraces a series of values, many originating from greater awareness of one’s position of dependency and discontent with the current system (Laldjebaev et al., 2015). For energy sovereignty to be more than a mere aspiration, technologies need to be further developed to facilitate energy production at a small scale and reduced costs. Over the last three decades we can witness enormous improvements. Independent production systems are becoming increasingly efficient, sustainable and financially accessible (Kaundinya et al., 2009; Lahoud, 2018). At the same time, information on small-scale production systems is becoming more popular, accessible in diverse media formats and widely shared through social media (Skjølsvold et al., 2018).

Access to technology and information empowers people and thereby leads to a stronger demand for change. Here the concept of energy sovereignty provides a strong umbrella term that allows people of vastly different interests and motivations to join forces under the same discourse. Energy production systems that are built on autonomous modules allow their users to gain independence from major energy corporations and secure energy under adverse conditions. As such, energy sovereignty is not only of general interest to the average citizens, but also appeals to the specific interest of particular groups, such as survivalists, environmentalists who oppose the massive burning of fossil fuels, and those living in areas prone to natural disasters, such as hurricanes and earthquakes. Therefore, a major advantage of the concept is that it can be supported by groups who otherwise are radically different and would not come together to voice their demands.

## Realising Energy Sovereignty

While the sovereignty ideal has been applied to other sectors, such as data and media (Hummel et al., 2021; Reilly, 2016), we will base our examination on the demands specified for agriculture as they have had the strongest influence in and outside academia (Edelman, 2014). The main advocacy for food sovereignty is done by Via Campesina, the largest peasant organisation of the world, composed of over 180 member organisations from more than eighty countries (Via Campesina, 2018), constituting the largest and most diverse non-governmental organisation seeking alternative production models based on popular control of food production and distribution channels (Menser, 2008). According to Via Campesina, food sovereignty constitutes a series of demands. We have analysed the different demands listed in the Food Sovereignty Declaration that was elaborated during the 1996 United Nations Food Summit held in Rome (Via Campesina, 1996). This declaration has been the most influential on food sovereignty and one of the most notable collective efforts to defend the right to self-determination (Patel, 2009). The declaration consists in seven key demands (i) the recognition of food as a human right, (ii) an agrarian reform for food sovereignty, (iii) the protection of natural resources, (iv) the reorganisation of food trade, (v) ending the globalisation of hunger, (vi) social peace, and (vii) democratic control. The preamble of this Declaration and later meetings organised by Via Campesina (Nyéléni Forum for Food Sovereignty, 2007) explicitly state the importance of recognizing the role of women for food sovereignty and their right to access the means to produce food and to employ and develop their skills. We therefore decided to include “equal opportunity and recognition” as an eight demand in our analysis to highlight gender issues.

In Table 1 we list these eight demands of food sovereignty. In each of these demands we have identified the underlying core ethical values by analysing the text of the Declaration (Via Campesina, 1996), while relying on academic literature and particularly on documents from later meetings of the farmers’ organisations to clarify ambiguities. The values “accessibility” (access), “sustainability” and “peace” are directly referred to in the Declaration. The value “recognition” was derived from the demand for land work needing to be “sufficiently valued”, “stewardship” was associated to the “right to sustainable management”, and “transparency” and “self-determination” are leant on “the right to honest, accurate information and open and democratic decision-making” (Via Campesina, 1996). We subsumed under “empowerment” the different demands for capacity-building and access to the means to produce food in the Declaration. In the 2007 meeting, the term “power” in relation to struggles and the capacity to influence and being influenced is one of the most frequently used terms (Nyéléni Forum for Food Sovereignty, 2007). “Resilience” was chosen as the value best representing the different demands to resists external economic, political and environmental pressures which may lead to supply shortages. While this value cannot be found as such in the 1996 declaration, it plays an important role in the meeting a decade later (Nyéléni Forum for Food Sovereignty, 2007). The value “gender justice” was chosen to represent the different demands for more opportunities for women. Based on the demands of food sovereignty and the identified core values, we hypothesise the corresponding demands to realise such values for energy sovereignty.

**Table 1:** Comparison of food sovereignty and energy sovereignty

Food sovereignty (based on Via Campesina (1996))	Underlying value	Energy sovereignty
Food as a basic human right	Accessibility	Energy as a right (at least instrumental to secure other human rights, e.g. health, security)
Agrarian reform: Access to the means of production Development of skills Recognition Capacity-building	Empowerment Recognition	Energy reform: Access to the means of production Development of skills Recognition Capacity-building
Protecting natural resources	Stewardship Sustainability	Facilitate climate and environmentally friendly energy production
Reorganising food trade	Self-sufficiency	Shield local energy production from energy dumping from unsustainable sources
Ending the globalisation of hunger Regulation and taxation of speculative capital	Resilience	Implement measures that protect people from energy poverty and major price fluctuations
Social peace	Peace	Social peace
Democratic control Honest and accurate information Open and democratic decision-making	Transparency Self-determination	Democratic control Prevent energy misinformation Open and democratic decision-making
Equality of opportunity and recognition	Gender justice	Equality of opportunity and recognition

As energy is a prerequisite to secure a large number of basic goods, ranging nowadays from education to political participation and from healthcare to shelter, we can expect a similar desire to participate in energy production and governance as in the case with food. The production of both goods should be compatible with our core values. Under current levels of corporate influence in politics, including politicians complementing salaries with advisory jobs in corporations, community empowerment seems to be the only path towards sovereignty. Yet despite this overlap, we can expect that as technology develops, both sectors will continue to add demands to this list. For instance, we speculate that the more specifically energy systems are monitored, demands for privacy will increase as people become aware how closely their energy consumption patterns can be recorded and how much such data reveals. We proceed by discussing each of the values associated with sovereignty in our analysis.

### Accessibility

Given our common dependence on food and the catastrophic problem of world hunger, it is unsurprising to see the right to food as the first demand in the declaration of Via Campesina. As we are highly dependent on energy for our needs, safety and well-being, we can rightfully claim that energy accessibility is a core value, as people have a fundamental interest in having a continuous energy supply at their disposal. In general terms, rights to access resources that are crucial for participation as equals in society count with a long history in political philosophy. A right to access the means to secure one’s subsistence has been defended for over four centuries in philosophy (Mancilla, 2019). In the more recent literature, particularly in relation to the human rights to food and to health, we can distinguish between the need for fair prices (accessibility), the continuous supply (availability) and the social adequacy of the products and production processes (De Schutter, 2011; Hassoun, 2020). Let us discuss in detail the first two elements, leaving the third element for the section on stewardship and sustainability to minimise overlaps.

Normatively, the right to access gains much more strength when energy becomes a necessity to secure and sustain life (Day et al., 2016). In some regions of the world access to energy is necessary to make a shelter liveable, for example to provide sufficient heating in the winter. Personal situations may create special needs. Older adults, small children, women during late pregnancy and sick people are more vulnerable to extreme temperatures. People with certain medical conditions that need life-sustaining or life-prolonging machinery are also in need of energy. We generally require energy to produce and prepare essential goods such as food, medicines and shelter. Insufficient access to safe, efficient and affordable energy for temperature regulation (heating or cooling), hygiene and food preparation is referred to as energy poverty (Lahoud, 2018). In addition, we have special energy needs that emerge due to the way we have structured social life: energy to engage in productive activities to secure livelihood and to communicate across long distances. In densely populated areas we need energy to provide sufficient illumination to discourage criminal activities and maintain law and order. This preliminary list already reveals that what constitutes a basic need and those needs to live a sufficiently flourishing life is blurry. Furthermore, we should also note that a human rights discourse demands the fulfilment of needs that go beyond securing basic life-sustaining needs. Energy is required to sustain basic democratic rights (publish and access information, organise) and to assist people to flourish in a number of ways (enjoy cultural life and participate in some games) (Hillerbrand, 2018). Society may also choose to make access to energy a protected good nobody ought to be excluded from, without reference to any particular use. For such reasons, access to energy is among the key indicators in the Sustainable Development Goals (Vera & Langlois, 2007). The central role of energy for human well-being have led some scholars to even consider energy as a human right (Ariza-Montobbio & Olarte, 2021; Dell’Anna & Menconi, 2016).

Similarly as with food, we may choose to temporarily abstain from using energy systems. Yet especially in our social context, such a decision needs to be voluntary. To allow people to freely participate in cultural and political life without moral hesitations, energy production needs to be consistent with people’s core values.

Expanding access by reducing or subsidising prices has a direct effect on the type of energy to be produced. Insisting on low prices may obligate governments to exploit cheap but socially and ecologically undesirable energy sources. Fortunately, technological innovation and environmental advocacy may hinder such outcomes.

It will have to be an issue of social consensus, taking in consideration the availability of natural resources, technological solutions and financial assets, to settle the question of when access is sufficiently secured and when to prioritise efficient use (Mitcham & Rolston, 2013).

### Empowerment and Recognition

Food sovereignty advocates demand an extensive agrarian reform to access the means of production: water, seeds and land, as well as training programs to make best use of these skills. Furthermore, it demands that society acknowledges the hard effort of those producing food – without recognition there is no respect for farmers and their work, and an incentive to continue farming is missing (Nyéléni Forum for Food Sovereignty, 2007).

Demands for a large-scale reform can be also seen in the energy sector. In general terms, sovereignty comes with a strong demand to gain power. Empowerment involves two steps. First, empowerment will have to come with strong initiatives to build capacities. It has been argued that a holistic approach to building capacities in the energy sector is needed. This requires (i) human resources (skills, knowledge, leadership), (ii) institutional resources for governance and management, (iii) knowledge resources (databases, traditional knowledge systems), (iv) community resources (social cohesion, networks), (v) physical resources (technology, roads), (vi) cultural resources (values, land ethic), (vii) natural resources (land, water), and (viii) financial resources (Rakshit et al., 2018). Second, energy policies need to give people the opportunity to use their resources and skills to contribute to their local energy system. Empowerment and the development of skills is valued for both instrumental and intrinsic reasons (Timmermann, 2018).

Having at a local level some liberty in drafting energy policies can help smaller communities to implement local solutions by making best use of local resources, such as wind, water streams, and on site expertise, and adapt energy production to local consumption patterns (Ariza-Montobbio & Olarte, 2021). It can tap from individual and group efforts in small-scale energy production and network them with others to sustain regional systems. Such policies can incentivize the independent production of clean energy and facilitate selling overproduction to adjacent energy grids. Participative policies can thereby empower people to do their share in establishing energy sovereignty. It also allows communities to be a role model in terms of sustainable energy production and self-sufficiency (Ariztia & Raglianti, 2020).

Empowerment at the organisational level allows citizens to build alliances to collaborate in responsible use and civil monitoring. This requires the development of capacities for political organisation and advocacy (Timmermann et al., 2018). Sovereignty cannot be achieved without the effective participation and meaningful contributions of a high threshold of citizens. Energy policies need to allow people to take independent action at the individual and community level to produce energy using renewable resources. Empowerment needs to go beyond reaching self-sufficiency at home. People need to have the power to change the energy systems they depend upon (Menconi et al., 2016).

An additional element within capacity-building includes learning about energy saving techniques, technologies and policies. Reducing demand is a first step to reduce dependency and environmental impact.

Similarly to the energy justice discourse (Jenkins, 2019), advocacy in sovereignty movements has included a strong demand to recognize people as possible solution providers and cooperation partners. Discrimination needs to be fought and participative policies favoured. Energy policies that recognize citizens as cooperation partners seek to empower them, incentivize the development of small-scale energy production systems and establish a legal framework that makes independent energy production attractive. Moreover, historical discrimination needs to be actively confronted to ensure the participation of underrepresented groups of people.

### Stewardship and Sustainability

The protection of natural resources is another element within the demands set forward in the declaration on food sovereignty. While all farmers are dependent on key ecological services, such as pollination, those who practice methods of ecological intensification have a higher stake in the protection of natural resources (Tittonell et al., 2016). Furthermore, farmers may not only value protecting the natural environment for instrumental reasons, but may also value nature for intrinsic reasons. For most farmers, the land they work is not only a workplace, but also their living space, which often leads to develop as stewards strong ties to the land and an appreciation of the local flora and fauna (Berry, 1977/2015).

Sustainability can be understood as a core value in the sense that people want a continuity in energy production without major drawbacks and unjustly imposing burdens on others. A full assessment needs to acknowledge that sustainability has a social, economic and environmental dimension (Werkheiser & Piso, 2015). Sustainability demands both to remove harmful incentives and internalise negative externalities. In addition, safeguards need to be in place to ensure that majority rule decisions do not have the effect of marginalising and overburdening minorities or the interests of future generations.

The protection of natural resources often requires active involvement, not only abstaining from interfering. Stewardship therefore emerges in this context as a central value. Appeals to sovereignty in general, including energy sovereignty, are inherently place-based practises with the goal of improving community well-being (Schelly et al., 2020). Gaining energy sovereignty would allow people to act as good stewards and direct energy policies towards the well-being of their community and the local environment.

In terms of social sustainability, some energy production systems are clearly far worse regarding work safety and environmental hazards than others. The coal industry has a notorious overall bad impact, considering health risk for workers in coal mines and effects on air quality for neighbourhood communities (Pouran, 2018).

Some exhaustible resources, like fossil fuels, can be put to much better use by people in the future than the use we give them now, which consists mainly in burning them (Singer, 2004). Another problem is the failure of our generation in internalising the negative externalities of our energy production. The costs of cleaning up pollution, handling nuclear waste, reforestation and land recovery, and adapting to climate change will largely be paid by future generations. The massive effect of these negative externalities will be hard to compensate for with technological fixes and therefore require urgent mitigation.

We can observe an increasing social demand to make energy policies more compatible with the interests of future generations. The engineering community, through the Report on Engineering for Sustainable Development, emphasises the important role and responsibility of engineers in reducing climate change emissions and improving the resilience of energy systems (UNESCO, 2021).

### Self-Sufficiency

Food sovereignty advocates demand to have the freedom to protect local markets and production systems from unsustainable business practises and subsidies. A major concern is the practice of food dumping of surplus produced in highly subsidised farmlands, particularly in the United States and the European Union. The sporadic availability of food below local production costs impedes local farmers to make a living by providing food to their communities (Nyéléni Forum for Food Sovereignty, 2007). Therefore, it hinders communities to determine how far they want to be self-reliant, as they cannot sustain their own food networks under the given market conditions (Agarwal, 2014). When imported food ceases to become available at low prices or cannot be delivered due to conflicts or natural catastrophes, the local population is at risk of hunger (Marrero et al., 2021). A reorganisation of food trade should allow people to establish the conditions that facilitate local food production and thereby become self-sufficient, in the sense of not being a net food importing country.

We can witness similar concerns in the energy sector. The availability of cheap energy such as fossil fuels and nuclear energy, whose true costs often remain hidden and are highly subsidised, makes it difficult to establish energy systems that are environmentally sustainable and do not oblige states to cooperate with countries with a poor human rights record (Patel & Moore, 2017). Economic discourses that blindly defend benefits of economies of scale and centralised production may also impede the establishment of policies that incentivize the development of local energy production facilities (Castán Broto, 2017).

Large investments in technological innovation are continuously allowing to improve technologies for generating energy at a small-scale, particularly as an effort to reduce carbon emission by incentivizing the expansion of renewable energy use. There are also some notable advancements in the capacity of batteries to store such energy.

### Resilience

Food sovereignty advocates demand an end of the globalisation of hunger. It is argued that financial speculation through the commodification of food have led to enormous price fluctuations and wasteful uses of food crops, with detrimental effects for people and the environment (De Schutter, 2017). People need a continuous supply of diverse foodstuff. Henceforth, later meetings organised by Vía Campesina have identified in community resilience and increasing resilience by working with nature central elements for food sovereignty to better absorb external shocks that affect food availability (Nyéléni Forum for Food Sovereignty, 2007). For this reason, we focus on resilience as the main value behind these demands. This broader interpretation also allows us to better explore the implications for the energy sector.

Resilience is becoming again a morally relevant value (Kolers, 2016). Disruptions in energy production are likely to occur due to extreme weather events caused by climate change and the spread of dangerous diseases. At the same time, we have become more dependent on continuous energy supply and particularly the most vulnerable, that is the poor with medical conditions, are the least able to absorb supply shocks. Energy supply needs to be continuously secured at least at a basic level. Power cuts can spoil a number of medicines and foodstuffs that need refrigeration, as well as distort essential services (e.g. transportation) (Erickson & Jennings, 2017). The way social life is designed, especially in urban areas, has made people extremely dependent on the continuous availability of energy. Dependence on unreliable energy sources greatly affects productivity and well-being.

Resilient system have been examined under different “4Rs”, for example “resistance, redundancy, response, and recovery” or “robustness, redundancy, resourcefulness, and rapidity”, distinguishing between the capacity to absorb stresses without disruptions and the capacities to adapt, respond and recover from disruptive stresses (Panteli & Mancarella, 2015). These characteristics can be achieved through an energy production mix that considers a technological diversification of the energy matrix with local and complementary renewable energy resources tailored to the different seasons and climate zones (Jurasz et al., 2020). The interconnection of energy systems needs to absorb production deficits systems without the risk of creating additional hazards, such as high voltage fluctuations (Ayele et al., 2018). The different production modules need to be diverse enough to accommodate shortfalls in inputs. These can be due to climate patterns (e.g. droughts and floods on hydroelectric facilities), extreme events (e.g. volcano eruption ashes on solar panels) and geopolitical changes (e.g. fuel delivery blockades). Energy producing individuals and communities need to recognize that despite their own self-sufficiency they are still dependent on goods produced under vulnerable energy systems. The establishment of resilient systems requires cooperation between the different producers and consumers to fix vulnerable elements.

To improve the resilience of energy systems, it is also crucial to assess the long-term effect of fiscal instruments in view of their economic sustainability (Wall et al., 2019). Some international capital flows are connected to vested interests and have been configuring the energy infrastructure creating path dependence, technological lock-in and long-term debt (Burchardt & Dietz, 2014). Subventions need to help build up desirable industries and have to be removed from areas that produce social and environmental hazards (Goldthau & Sovacool, 2012). Additional tolls need to be collected to have the resources to compensate those who suffer adverse effects. Regulations need to be implemented to avoid that financial speculations have an undesirable effect on energy production and society. For instance, a strategy pursued since the early 1970s oil crisis in Denmark has been to reduce dependency from imported oil by investing heavily in energy diversification and giving heat and electricity production at district level a central role to improve self-sufficiency (Sovacool & Martiskainen, 2020). By giving more power to lower levels of public administration, citizens have a higher chance to influence energy decisions and align them to long-term community well-being.

### Peace

As many conflicts play out in rural areas, where often central governments have little control, farmer organisations have listed social peace within their central demands for food sovereignty. Conflicts impede carrying out and maintaining long-term projects and lead to the losses of lives, and therewith knowledge, skills and workforce. Food shortages are a major source of conflict and social unrest (Holt-Giménez & Patel, 2009).

Over the last decades, control over energy resources, particularly oil fields, has been the central driver for numerous wars and occupations (Wenar, 2015). Cheap oil has come at an enormous price in terms of human lives, suffering, and environmental destruction, as has been amply documented in Nigeria (Onwuazombe, 2017). Moral decency obliges us to dissociate from such energy production systems and not contribute to their continued existence by remaining dependent on them. Moreover, energy resources are often the cause of conflicts when sovereignty claims over such resources is not recognized or disputed (Redgwell, 2021), or when neighbouring nations cannot find a peaceful solution over resources that are between national borders, such as natural gas deposits.

It is also worrying with what force large-scale energy projects are being imposed against the will of local communities (Temper et al., 2020). Especially in Latin America and Africa, we have witnessed the harassment and even killing of environmental activists who have fought such projects, as the well-known case of the murder of the indigenous leader Berta Cáceres (Arguedas Ramírez, 2018) and the execution of the writer Ken Saro-Wiwa (Bellow et al., 2018) show. In some cases, the newly available hydric resources resulting from dams have led to new violent conflicts as different groups attempt to control them (Pelayo Pérez & Rasch, 2020).

The strong impact nuclear energy has had in questions related to safety invites us to discuss this worry as a separate value related to peace. There are concerns that radioactive material can be weaponized, but also the dangers of nuclear fallout are ever present. Energy production comes with certain risks, for people, nature and electronic devices, which need to be minimised. Energy sovereignty is incompatible with dependency on systems that do not meet people’s values regarding safety and peace.

### Transparency and Self-Determination

Food sovereignty advocates demand democratic control over food and agricultural policies, by making explicit reference to being provided accurate information, and urging for transparency and democracy in decision-making processes. The central values that emerge from these demands are transparency and self-determination. There are two major discontents that have led farmer organisations to bring forward this demand: the imposition of certain technologies, such as GM seeds, and agrarian policies that give preference to large-scale production for export markets (Patel, 2009).

We can find similar requests among the population for more control over energy policies. The way energy is produced may touch firmly on people’s values and thereby it is important for people to have a say on the content and direction of energy policies. Yet similarly as in the food sector (Hospes, 2014), there is some debate at what level energy sovereignty should be sought at. Those who are strongly inspired by community-led efforts and the learnings from food sovereignty advocates, are more likely to define energy sovereignty as local communities deciding over energy policies without being dominated by the interests of corporations and political elites (Ariza-Montobbio & Olarte, 2021; Dell’Anna & Menconi, 2016). This interpretation would favour grassroots initiatives and efforts to decentralise energy production systems. Another interpretation argues for governmental control over energy resources (Barnes, 2014). Particularly countries with a colonial history, or who suffered aggressive foreign interventions, are open to discourses that claim to facilitate self-determination through state control or even the nationalisation of energy resources (Fitz‐Henry, 2015).

One of the main discontents about the energy sector concerns its environmental footprint. Energy systems may hinder the ability to live in a clean and diverse natural environment, free from pollution and the destruction of nature. The continuous dependence on energy may lead to an inner conflict as one becomes aware of how one indirectly contributes financially to states, rebel groups and companies that do not respect human rights and nature. Energy politics that make the citizenry dependent on purchasing energy from entities that repeatedly violate human rights directly impact people’s ability to live a flourishing life while not contributing to the misfortune of others. Moreover, massive energy production projects may have a detrimental effect on a number of communities. Many dams in the tropical regions also serve as mosquito breeding sites facilitating the propagation of malaria and dengue, generate conflicts over the use of water resources, affect the characteristics of the river and generate methane emissions, among various other socio-economic impacts (De Faria et al., 2017). The demand for land to grow crops destined for the production of biofuels conflicts with the demand for access to land to grow food (Moreno & Mittal, 2008). Particularly when the development of energy production sites lacks transparency and public consultation, eventual problems, such as displacement and different forms of pollution, may lead to higher levels of resistance and discontent among the neighbouring communities (Borch, 2018).

As energy politics have such deep effects on people and the environment, it becomes only natural that people demand to be included in decision-making mechanisms and claim as a group a right to self-determination over policies that directly affect them (Pesch et al., 2017). To increase participation in decision-making is a central demand of procedural justice and a moral value in itself (Oosterlaken, 2015). Democratic participation is of course only meaningful if citizens have indeed the power to object to certain forms of energy production, such as nuclear energy. To give a voice to historically underrepresented and marginalised groups has been one of the main demands since the early development of environmental justice scholarship (Shrader-Frechette, 2002). By definition people need to participate in decision-making processes to enjoy sovereignty, and as mentioned earlier, effective participation requires knowledge and skills. To balance policies that aim at the production of energy with policies that are aimed to address other social interests we need to increase the public understanding of science, develop platforms to facilitate a dialogue, improve democratic decision-making mechanisms and facilitate access to information to allow for transparency.

The worrying expansion of anti-environmentalist populism and the craving for cheap energy urges us to link appeals to wider self-determination in energy politics with improving energy and environmental literacy. Populism accompanied by neo-extractivism has distorted the environmental narrative. In Latin America in the first two decades of the twenty-first century, populist governments have introduced large-scale hydropower in the national taxonomy as a clean energy source in the name of climate change, often without an adequate assessment of its socio-environmental impacts and allowing a concentration of power, investment, and energy production in the hands of a few. Many corruption cases related to the Panama Papers were related to hydroelectric projects (de la Torre, 2020; Ioris, 2021).

Self-determination will have to come hand in hand with self-constraint and responsible use (Cotarelo et al., 2014). To maintain energy sovereignty, people need to cooperate, for example by using less energy during cloudy days if electricity supply comes from photovoltaic installations. Observations from community controlled solar energy systems reveal that people are willing to adjust their energy use to supply variations (Ariztia & Raglianti, 2020). In cases where energy can be obtained abundantly at low costs, it will take substantial public education efforts so that people do not use energy wastefully. But, as we can observe from efforts to reduce wasteful food habits, people are open to change their behaviour on the basis of moral arguments in favour of sustainability (Singer, 2004).

Increasing the democratisation of energy systems does not necessarily have to come with a nationalisation of such systems. Yet for governments to be able to exert sufficient pressure over non-complying energy companies, it will be inevitable to establish regulations limiting the size and supply-chain control of energy companies through antitrust laws. To maintain sovereign control, people need to make sure their current production facilities do not place people in the future in a situation of dependency and vulnerable to extortion. Long-distance pipelines for oil and gas that only pay off after more than a decade of use are a well-known example of dependent energy systems (Zhang et al., 2019).

### Gender Justice

For food sovereignty advocates, issues of gender justice concentrate on access to food for women and girls, equal rights to own land and access water, to develop and use skills, to lead as a woman a farm, and the protection and recognition of labour done primarily by women, such as the conservation and further breeding of heritage varieties (Shiva, 2009). A major worry is that the imposition of new commercial seed varieties may deprive women of an important source of recognition, interaction and power, without offering something that will compensate them for this loss (Nyéléni Forum for Food Sovereignty, 2007).

To achieve gender justice still massive work needs to be done towards improving equality of opportunity in gaining and freely exercising technical and political expertise. As the energy sector requires a high percentage of jobs with certified training and education (Rakshit et al., 2018), it is crucial to ensure that women and girls have adequate opportunities to go from early on to educational programs that will allow them to participate in the energy sector as equals, particularly as technicians and engineers, areas in which they are still underrepresented (UNESCO, 2021). This also requires to abolish discriminatory politics and practices that impede or endanger the possibility for women to work in the energy sector. Industries with a poor record of ensuring equality of opportunity for women, such as offshore oil platforms, have managed in individual cases to improve their standing by actively confronting gender issues (Ely & Meyerson, 2010).

Particularly in the rural Global South, we have to recognize that women and girls are overwhelmingly burdened with collecting firewood. Even in the rare cases when such arrangements follow an equitable division of work, we still need to ensure that girls and women have adequate opportunities to improve energy efficiency and use. Initiatives where women teach other women how to build more energy-efficient stoves can greatly improve lives in traditional rural households (Troncoso et al., 2007). A lesson from agricultural technology assessment is to pay attention that innovation does not deprive women of the last excuses they had to leave the house and interact with others (Shiva, 2009). Measures to empower women need to be taken to avoid unintended negative consequences of innovation.

## What Can We Learn from the Food Sovereignty Movement?

The sovereignty discourse leaves many open questions when we try to apply it to energy. For instance, as mentioned with regard to the food sovereignty movement (Hospes, 2014), we need to be more specific in regard to the level we want to achieve energy sovereignty. Sovereignty can be claimed at a community, provincial, national or regional level. In agriculture, efforts to achieve sovereignty at a community level have been particularly successful, as they build on community cohesion, shared values and a similar sense of urgency. Furthermore, successful community initiatives serve as role models that are often replicated by neighbouring communities. Community cohesion needs to be recognized as a major social capital for alternative energy production systems and complemented with efforts to improve energy literacy. This is well-reflected in community-based renewable energy projects. Here, in partnership with the local government and private enterprises, a group of citizens undertakes an energy generation project, where all parties are owners and beneficiaries of the project, satisfying local demand and selling surplus to the national grid (Dütschke et al., 2019).

When energy sovereignty is sought at a community level, this may bring issues of social justice as some communities are likely to be much better endowed to reach self-sufficiency. Geographic, technological, social and environmental factors may create major barriers towards achieving sovereignty. Collaborative multisectoral networks need to be built to compensate for such differential capacities to attract investments, share resources and geographic advantages, in order to reduce costs, improve efficiency, distribute risks and elaborate together the most environmentally sustainable energy production systems (Olivadese et al., 2021) based on renewable energies such as solar, wind, biomass, geothermal, among others. However, when local communities are not prepared for the adoption of technologies, either by missing adequate information or not having been able to strengthen local capacities, it will be difficult to introduce the needed technologies (Caramizaru & Uihlein, 2020). In an interconnected world, energy sovereignty may sometimes stumble on the effective performance of its weakest link.

A special issue for the energy sector concerns modularity. Energy supply requires a well-functioning socio-technical system, composed of a series of single-standing elements that interact with each other and can be adapted to changes at the global as well as local level and incorporate forthcoming technological innovations. Modular systems have great versatility by promoting system resilience and adaptability. They allow to increase or to reduce the installed capacity and to replace modules that fail for new ones, avoiding the failure of the system as a whole. Modularity should ideally be composed of a diversity of technologies (e.g. solar, wind, biomass), creating modules that complement each other in times of deficit and surplus, where energy storage becomes an element of synergy and energetic, economic and environmental optimisation (Tronchin et al., 2018). Systems that complement each other can share surpluses when environmental conditions are favourable and assist adjacent systems who suffer from temporary short-falls. Modularity also facilitates the progressive realisation of energy sovereignty by allowing pioneering communities to take independent action and guide others with their example and experience.

Another important lesson we need to learn from the food sovereignty movement, is to pay attention that terms are not being redefined by those in power, as has happened with the definition of hunger by losing reference to the true caloric needs of those engaged in physical labour (Lappé et al., 2013). Such redefinitions also need to be taken seriously in the energy sector. At the time of writing, there is substantial outrage at plans by the European Commission to give nuclear energy and gas under certain conditions a green investment label (Strauss, 2022).

Lastly, energy sovereignty is achieved as a community. It has a public good character as everyone benefits from sovereignty once it is established. As a public good, sovereignty faces the traditional problem of free-riding. The more people fail to participate in establishing sovereignty, the less sovereign the energy system becomes. In this sense, sovereignty is closely related to participatory governance and democratic models (Szulecki, 2018). It is important to keep these characteristics in mind as the concept has been repeatedly co-opted to favour those who already have power.

## Concluding Remarks and Future Outlook

Realising sovereignty is a gradual process that needs to be progressively achieved and continuously defended. To advance in this stepwise process, people need to take advantage of the different developments concerning technological innovations, climatic changes, forms of organisation, information technologies and social theory on cooperation. People need to be receptive to new developments and acquire skills as individuals and as communities to adapt and make best use of innovations. Here attention needs to be paid to not only focus on the technological side, but also recognize the potential role the development of ethical concepts can have. The development of notions of solidarity and responsibility, and their effective transmission to the community may have a substantial effect on improving resilience of energy systems. As energy sovereignty can only be achieved as a community, building social cohesion and willingness to cooperate are as important as making best use of technological advancement. Energy decentralisation encompasses social, infrastructural and financial processes (Bosch & Schmidt, 2020). The required technological transformation is interconnected with capacity-building and participation in decision-making.

As demands for energy sovereignty increase, it becomes crucial that engineers incorporate the various values in this appeal in the design processes of new technologies. A values-sensitive design of energy systems needs to support such demands, and integrate as far as possible these values in the development and design processes. Furthermore, the repeated calls for wider public inclusion require to lift barriers to make educational and career programs in engineering more inclusive.

Due to the roots of sovereignty movements it is likely that certain issues will remain unaddressed. A value we had trouble fitting in this framework is privacy in smart-grids (cf. Jenkins et al., 2020). This is a concern that may require to include a new demand in the energy sovereignty discourse or be dealt with using a different ethical framework. Current work on data sovereignty suggests that citizen control over energy use data will soon be incorporated in energy sovereignty discourses (Bria, 2019).
